# Thyroid-related adverse events induced by immune checkpoint inhibitors

**DOI:** 10.3389/fendo.2022.1010279

**Published:** 2022-09-20

**Authors:** Alexandra Chera, Andreea Lucia Stancu, Octavian Bucur

**Affiliations:** ^1^ Victor Babes National Institute of Pathology, Bucharest, Romania; ^2^ Carol Davila University of Medicine and Pharmacy, Bucharest, Romania; ^3^ Brigham and Women’s Hospital and Harvard Medical School, Boston, MA, United States; ^4^ Viron Molecular Medicine Institute, Boston, MA, United States

**Keywords:** immune checkpoint inhibitors, cancer treatment, CTLA-4, PD-1, PD-L1, immune-related adverse events, endocrine, thyroid

## Abstract

Immune checkpoint inhibitors, namely anti-CTLA-4, anti-PD-1 and anti-PD-L1 monoclonal antibodies, have emerged in the last decade as a novel form of cancer treatment, promoting increased survival in patients. As they tamper with the immune response in order to destroy malignant cells, a new type of adverse reactions has emerged, known as immune-related adverse events (irAEs), which frequently target the endocrine system, especially the thyroid and hypophysis. Thyroid irAEs include hyperthyroidism, thyrotoxicosis, hypothyroidism and a possibly life-threatening condition known as the “thyroid storm”. Early prediction of occurrence and detection of the thyroid irAEs should be a priority for the clinician, in order to avoid critical situations. Moreover, they are recently considered both a prognostic marker and a means of overseeing treatment response, since they indicate an efficient activation of the immune system. Therefore, a multidisciplinary approach including both oncologists and endocrinologists is recommended when immune checkpoint inhibitors are used in the clinic.

## Introduction

The influence of the immune system over tumor development is of high importance, since the immune system can recognize and destroy tumor cells. This is not the case for every type of tumor: different mechanisms are involved in a process called immune escape, helping malignant cells to avoid being killed (1). Our immune system could also respond against healthy tissues, leading to autoimmunity, which is normally prevented through activation of inhibitory pathways known as immune checkpoints. When these pathways are not properly regulated, immune escape of cancer cells may occur (2).

Immune checkpoint inhibitors (ICIs) have emerged as a new class of drugs used for in treatment of various types of malignancies, since their first U.S. Food and Drugs Administration (FDA) approval, in 2011 of ipilimumab (3, 4). Anti-cytotoxic T-lymphocyte-associated antigen 4 (CTLA-4) monoclonal antibodies (e.g. ipilimumab), anti-programmed death 1 (PD-1) monoclonal antibodies (e.g. pembrolizumab, nivolumab, cemiplimab) and anti-PD-1 ligand (PD-L1) monoclonal antibodies (e.g. avelumab, atezolizumab, durvalumab) are the types of ICIs used at the moment (5). They have improved overall survival for patients suffering from neoplasms, such as melanoma, non-small cell lung cancer (NSCLC) and renal cancer. However, ICIs are also the cause of specific adverse reactions known as immune-related adverse events (irAEs) (6). IrAEs are usually phenotypically similar to autoimmune conditions, even though it is not certain that the underlying mechanisms are similar. Some predisposing factors that might lead to irAE are genetic and environmental, or a combination of both (7).

Common irAEs associated with ICI therapy include thyroid dysfunctions, both hypothyroidism and hyperthyroidism or thyrotoxicosis, especially while administering anti-PD-1 or anti-PD-L1 antibodies. Therefore, this form of cancer treatment challenges both oncologists and endocrinologists and it should involve a multi-disciplinary approach (8).

## Immune checkpoint inhibitors: Mechanism of action

The therapy involving modulation of/tampering with the immune system in order to treat malignant tumors is called immunotherapy. It involves cancer vaccines, an oncolytic virus, a bi-specific T-cell and ICIs (9). ICIs have emerged in the last decade, changing the outlook on treatment for at least 17 different types of cancer (3).

Immune checkpoints are responsible for self-tolerance, while inhibitory checkpoint molecules such as CTLA-4, PD-1 and its ligands PD-L1 and PD-L2, regulate this process. Inflammation is said to upregulate these molecules through interferon gamma. Antibodies targeting CTLA-4 or PD-1 and its ligands ensure the enhancement of immune reactions against malignant cells, while also promoting autoimmunity (3, 6, 9).

**Figure 1:**
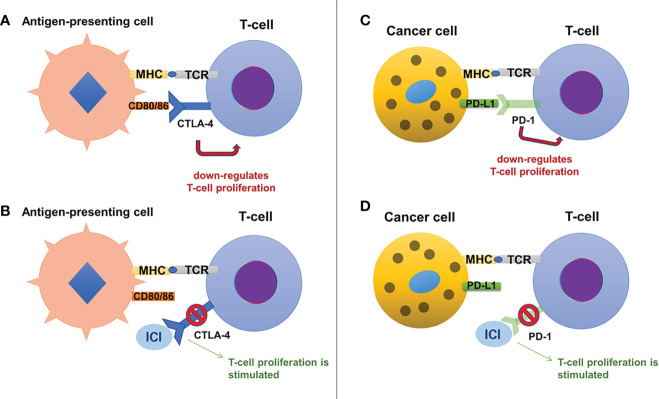
The mechanisms of action for immune checkpoint inhibitors include the CTLA-4 pathway (on the left, panels **(A)** and **(B)**) and the PD-1/PD-L1 pathway (on the right, panels **(C)** and **(D)**). Normally, CTLA-4 is present on the surface of T-cells and by binding to its ligands, CD80 and CD86 (expressed on antigen-presenting cells), it exerts an inhibitory effect on the T-cell proliferation **(A)**. Anti-CTLA-4 antibodies (a variety of ICIs) bind to CTLA-4 and block its inhibitory effects, stimulating T-cell proliferation **(B)**. Similarly, T-cells express PD-1, which binds to its ligand, PD-L1, present on cancer cells, suppressing T-cell proliferation **(C)**. Anti-PD-1 antibodies bind to PD-1 blocking its inhibitory effects and stimulating T-cell proliferation **(D)**.

**CTLA-4** (also known as CD152) is a glycoprotein expressed by CD4+ and CD8+ T-cells (10), acting as a negative regulator for T-cell activation (11). This inhibitory effect is based on its similarities to CD28, which enable binding of CTLA-4 to B7 glycoproteins (B7.1 or CD80 and B7.2 or CD86) with 20-fold higher affinity than CD28 (11, 12). This results in limitation of activated T-cell proliferation (8), promoting the immune escape of cancer cells (see **Figure 1**).

**PD-1** (also known as CD279) is mainly expressed on the activated CD8+ T-cells (13), although it is also present on the surface of macrophages, dendritic cells, B-cells, and while it is also similar to CD28, it binds to its specific ligands: PD-L1 (expressed on immune cells, hepatocytes, pancreatic islet cells, endothelial cells, myocytes, thyroid cells and many other cells, including various tumor cells) and PD-L2 (only expressed on macrophages and dendritic cells) (12). The inhibitory effect on T-cell activity is obtained through binding of PD-1 to its ligands, which results in a decrease of glucose uptake and gluconeogenesis of T-cells and apoptosis, while also interrupting the co-stimulatory pathway of CD28-CD80/86 (11, 12). Only regulatory T-cells will have an increased survival, as they have the ability to suppress cytotoxic CD8+ T-cell proliferation, in favor of immune escape of cancer cells (11, 14). The PD-1 ligands seem to be present mostly in inflammatory settings as they are strongly regulated by interferon gamma (15): this could be a reason why chronic inflammation which surrounds tumors limits the destruction of cancer cells (16). Also, the high expression of PD-L1 which promotes immune escape was linked with greater tumor aggressiveness (17).

Considering the fact that CTLA-4 and PD-1 regulate different stages of the immune response (CTLA-4 regulates the early stages of T-cell activation, while PD-1 is expressed after the T-cells are activated) and that they exert their actions at different sites (draining lymph nodes for CTLA-1 and tumor microenvironment for PD-1 and its ligands), it is understandable that their effects and adverse reactions are different (18–20). PD-1 inhibitors seem to have a more specific effect, associating less severe adverse reactions (20–23).

Both CTLA-4 and PD-1 are also involved in development of autoimmune disorders. CTLA-4 seems to have a role in the development of Graves’ disease (24) and immune dysregulation syndrome, through autosomal dominant mutations (25). PD-L1 expression is increased in Hashimoto’s thyroiditis (26), while PD-1 blockade might stimulate B-cells to produce thyroid autoantibodies and thyroid dysfunction, especially in patients with higher baseline levels of these thyroid antibodies prior to the treatment (1, 27, 28).

The importance of prognostic markers and routine laboratory investigation before deciding to use a specific cancer treatment option has been previously established (29). Potential prognostic markers with either proven efficacy or in-line for future studies are: tumor mutational burden, PD-L1 expression, fraction of copy number alteration, human leukocyte antigen (HLA)-I evolutionary divergence, the loss of heterozygosity status in HLA-I25, microsatellite instability, body mass index, sex, blood neutrophil-to-lymphocyte ratio (NLR), tumor stage, immunotherapy drug agent, age, cancer type, whether the patient received chemotherapy before immunotherapy, blood levels of albumin, platelets and hemoglobin (the last three providing information about the presence of tumor-promoting inflammation), other molecular features of the tumor immune microenvironment (infiltration levels of myeloid cells or lymphocytes), composition of the microbiome, T-cell receptor diversity, mutations associated with resistance to ICIs and the expression of ADORA1 or p62 (30–47).

Currently, the main FDA-approved prognostic marker for ICI response is the tumor mutation burden, which measures the number of mutations present in the tumor, proportional to the number of neo-antigens and to the probability of triggering a T-cell response. It performs well on its own, but the efficacy can be increased by associating the detection of PD-L1 expression (48, 49) or NLR (33). Regarding PD-L1 expression, it has been hypothesized that the presence of tumor infiltrating lymphocytes might increase its accuracy in predicting the ICI treatment response (35). NLR is an accessible marker of systemic inflammation, negatively correlated to cancer prognosis and response to immunotherapy (32).

## FDA-approved immune checkpoint inhibitors

The first ICI approved by the FDA for treating metastatic melanoma (2011) was ipilimumab, an anti-CTLA-4 monoclonal antibody, and it was followed by the FDA-approval of three anti-PD-1 (pembrolizumab, nivolumab, cemiplimab) and three anti-PD-L1 monoclonal antibodies (atezolizumab, durvalumab, avelumab) (5). Along with ipilimumab, another anti-CTLA-4 antibody known as tremelimumab has entered clinical trials, but its antineoplastic activity was low while it was administered as a single drug, and it has also shown substantial side effects (the incidence of thyroid disorders is 0-5.2%). However, its association with durvalumab for treating mesothelioma, melanoma, NSCLC, gastroesophageal or colorectal tumors could be promising, even though some studies have not shown a substantial benefit added by tremelimumab compared to durvalumab alone (50–52). A phase 3 clinical trial (CheckMate 037) has shown that anti-PD-1 antibodies have both higher efficacy and a superior safety profile compared to ipilimumab (53). Another association of ICIs considered to be successful is the combination of ipilimumab with nivolumab, which has shown 57% intracranial central nervous system response for patients suffering from melanoma brain metastases (54, 55). ICIs can also be associated with chemotherapy agents to treat certain types of cancer: in 2019, FDA has approved the combination of atezolizumab and paclitaxel for treating patients with advanced metastatic triple negative breast cancer expressing PD-L1, without previous systemic treatment for metastatic disease (56, 57). Also, radiation therapy might be useful alongside ICI treatment, as it can sensitize melanoma brain metastases to ICIs (55, 58).

While most of the studies about ICIs involved solid tumors, their promising effects have paved the way for treating hematological malignancies as well: nivolumab and pembrolizumab were approved by the FDA in 2016 and 2017 for the treatment of the relapsed/refractory Hodgkin’s lymphoma (5, 59, 60).

The anti-CTLA-4, anti-PD-1 and anti-PD-L1 antibodies belong to different categories: ipilimumab, atezolizumab, durvalumab and avelumab belong to the IgG1 subclass, activating the classical complement pathway and initiating antibody-dependent cell-mediated cytotoxicity (ADCC), while pembrolizumab, nivolumab and cemiplimab are part of the IgG4 subclass, which is not able to activate the classical complement pathway and has a lower potency in inducing ADCC (10).

**Ipilimumab (Yervoy^®^)** is a CTLA-4 inhibitor belonging to the IgG1 subclass, approved by the FDA in March 2011 (4, 9). It is used for the treatment of melanoma, renal cell carcinoma and colorectal cancer (13), while also being responsible for development of irAE, usually after 9 weeks of therapy (hypophysitis, hypothyroidism, hyperthyroidism are the most frequent) (9, 61, 62).

**Pembrolizumab (Keytruda^®^)** is a PD-1 inhibitor belonging to the IgG4 subclass, approved by the FDA in September 2014 (9, 63). It is used for the treatment of melanoma, small-cell lung cancer (SCLC), NSCLC, Hodgkin’s lymphoma, squamous cell carcinoma of the head and neck, urothelial carcinoma, gastroesophageal and colorectal cancer, primary mediastinal large B cell lymphoma, hepatocellular carcinoma, Merkel cell carcinoma, cervical and renal cell cancer (13). The most frequent irAEs induced by pembrolizumab are hypothyroidism and hyperthyroidism (64), which have a particular immune phenotype demonstrated by studies: elevation of CD56^+^CD16^+^ natural killer (NK) cells along with decrease of CD56^br^CD16^-^ NK cells and CD14^+^HLA-DR^lo/neg^ monocytes. PD-1 is not expressed on T-cells from pembrolizumab-induced thyroiditis, which supports a T-cell mediated mechanism, rather than a B-cell mediated one (65).

**Nivolumab (Opdivo^®^)** is a PD-1 inhibitor belonging to the IgG4 subclass, approved by the FDA in 2015 as a second-line treatment for NSCLC (grades IIIB and IV) (66, 67). Currently, it is used for the treatment of melanoma, SCLC, NSCLC, Hodgkin’s lymphoma, squamous cell carcinoma of the head and neck, urothelial carcinoma, microsatellite instability-high or mismatch repair deficient colorectal cancer, hepatocellular and renal cell carcinoma (10, 52, 68). The combination of nivolumab and ipilimumab is the most frequent cause of hypothyroidism (CheckMate 069 trial has shown an incidence of 17%) (9, 69, 70). Nivolumab-induced thyrotoxicosis shows similarities to autoimmune thyroiditis (predominance of CD8^+^ T-cells), while also showing granulomas, destruction of thyroidal follicles and chronic thyroid lymphocytic inflammation, which are not present in autoimmune thyroiditis (71).

**Cemiplimab (Libtayo^®^)** is a PD-1 inhibitor belonging to the IgG4 subclass, approved by the FDA in September 2018, for the treatment of metastatic or locally advanced cutaneous squamous cell carcinoma not amenable to surgery or radiation (72, 73). Clinical studies are conducted for evaluating its efficacy in NSCLC, renal, ovarian and uterine carcinomas (74). It is responsible for the following irAEs: hypothyroidism (a research study has found an 8% incidence of hypothyroidism in a cohort of 59 patients treated with cemiplimab) (75), hyperthyroidism, type 1 diabetes mellitus (T1 DM), adrenal insufficiency and hypophysitis (76).

**Atezolizumab (Tecentriq^®^)** is a PD-L1 inhibitor belonging to the IgG1 subclass, approved by the FDA in 2016 for the treatment of urothelial carcinoma (77). It has also shown efficacy in SCLC, NSCLC, triple-negative breast cancer and metastatic squamous cell carcinoma of the head and neck (13, 78–81). It can induce hypothyroidism as irAE (64).

**Durvalumab (Imfinzi^®^)** is a PD-L1 inhibitor belonging to the IgG1 subclass, approved by the FDA in 2017 for the treatment of urothelial carcinoma and NSCLC (13, 82), while inducing hypothyroidism and hyperthyroidism as irAEs (64).

**Avelumab (Bavencio^®^)** is a PD-L1 inhibitor from the IgG1 subclass, approved by the FDA in 2017 for urothelial carcinoma, Merkel cell carcinoma and renal cell carcinoma (13, 83), while inducing hypothyroidism as irAE (64).

The information about the ICIs described above is synthetized in **Table 1**.

**Table 1:** Immune checkpoint inhibitors, cancer types for which they are used and the most frequently induced immune-related endocrine adverse events.

Drug name	Class	IgG subclass	FDA approval	Cancer types for which the drug is used	Frequent immune-related endocrine adverse events
Ipilimumab (Yervoy^®^)	Anti-CTLA-4 monoclonal antibody	IgG1	2011	Melanoma, renal cell carcinoma, colorectal cancer	Hypophysitis, hypothyroidism, hyperthyroidism
Pembrolizumab (Keytruda^®^)	Anti-PD-1 monoclonal antibody	IgG4	2014	Melanoma, small-cell lung cancer (SCLC), non-small-cell lung cancer (NSCLC), Hodgkin’s lymphoma, squamous cell carcinoma of the head and neck, urothelial carcinoma, gastro-esophageal and colorectal cancer, primary mediastinal large B cell lymphoma, hepatocellular carcinoma, Merkel cell carcinoma, cervical, renal cell cancer	Hypothyroidism, hyperthyroidism
Nivolumab (Opdivo^®^)	Anti-PD-1 monoclonal antibody	IgG4	2015	Melanoma, SCLC, NSCLC, Hodgkin’s lymphoma, squamous cell carcinoma of the head and neck, urothelial carcinoma, microsatellite instability-high or mismatch repair deficient colorectal cancer, hepatocellular, renal cell carcinoma	Hypothyroidism, hyperthyroidism
Cemiplimab (Libtayo^®^)	Anti-PD-1 monoclonal antibody	IgG4	2018	Cutaneous squamous cell carcinoma, NSCLC, renal, ovarian, uterine carcinomas	Hypothyroidism hyperthyroidism type 1 diabetes mellitus, adrenal insufficiency, hypophysitis
Atezolizumab (Tecentriq^®^)	Anti-PD-L1 monoclonal antibody	IgG1	2016	Urothelial carcinoma, SCLC, NSCLC, triple-negative breast cancer, metastatic squamous cell carcinoma of the head and neck	Hypothyroidism
Durvalumab (Imfinzi^®^)	Anti-PD-L1 monoclonal antibody	IgG1	2017	Urothelial carcinoma, NSCLC	Hypothyroidism, hyperthyroidism
Avelumab (Bavencio^®^)	Anti-PD-L1 monoclonal antibody	IgG1	2017	Urothelial carcinoma, Merkel cell carcinoma, renal cell carcinoma	Hypothyroidism

## Immune checkpoint inhibitors: Immune-related endocrine adverse events

The immunologic tolerance can be altered after the administration of ICIs, therefore the risk of targeting self-antigens is high (84). A new category of adverse events has emerged as a consequence of the augmented immune response - they are known as immune-related adverse events (6) and they are present in the majority of the patients treated with anti-CTLA-4 and anti-PD-1 antibodies (a clinical trial has identified 86% occurrence of irAEs after ipilimumab administration, 82% after nivolumab and 96% after combination therapy) (70, 85), the most frequent being the thyroid dysfunctions (86).

Common Terminology Criteria for Adverse Events (CTCAE) are used for grading the irAEs induced by ICIs. There are 5 grades of severity, grade 1 representing mild toxicity and grade 5 representing death caused by the adverse reaction (see **Table 2**) (87). CTLA-4 inhibitors are responsible for causing grade 3 or 4 irAEs in >34% of the patients, while PD-1 inhibitors induce grade 3 or 4 irAEs in 15% of the patients. ICIs combinations are the most frequent elicitors of severe irAES (58-68%) (70, 88–91). Nivolumab and pembrolizumab are considered to be more easily tolerated than ipilimumab in terms of irAE severity (6, 70, 92).

**Table 2:** Grading guidelines proposed by Common Terminology Criteria for Adverse Events (CTCAE) (applicable for immune-related adverse events induced by immune checkpoint inhibitors).

Grade 1(Mild)	Grade 2(Moderate)	Grade 3(Severe)	Grade 4(Life-threatening)	Grade 5(Death)
- asymptomatic or mild symptoms - only clinical or diagnostic observations - intervention not indicated.	- minimal, local or noninvasive intervention indicated - limiting age-appropriate instrumental activities of daily living.	- medically significant, but not immediately life-threatening - hospitalization/prolongation of hospitalization is indicated - disabling - limiting self-care activities of daily living.	- urgent intervention is indicated.	- death related to adverse events.

Adapted from reference (87).

**Figure 2:**
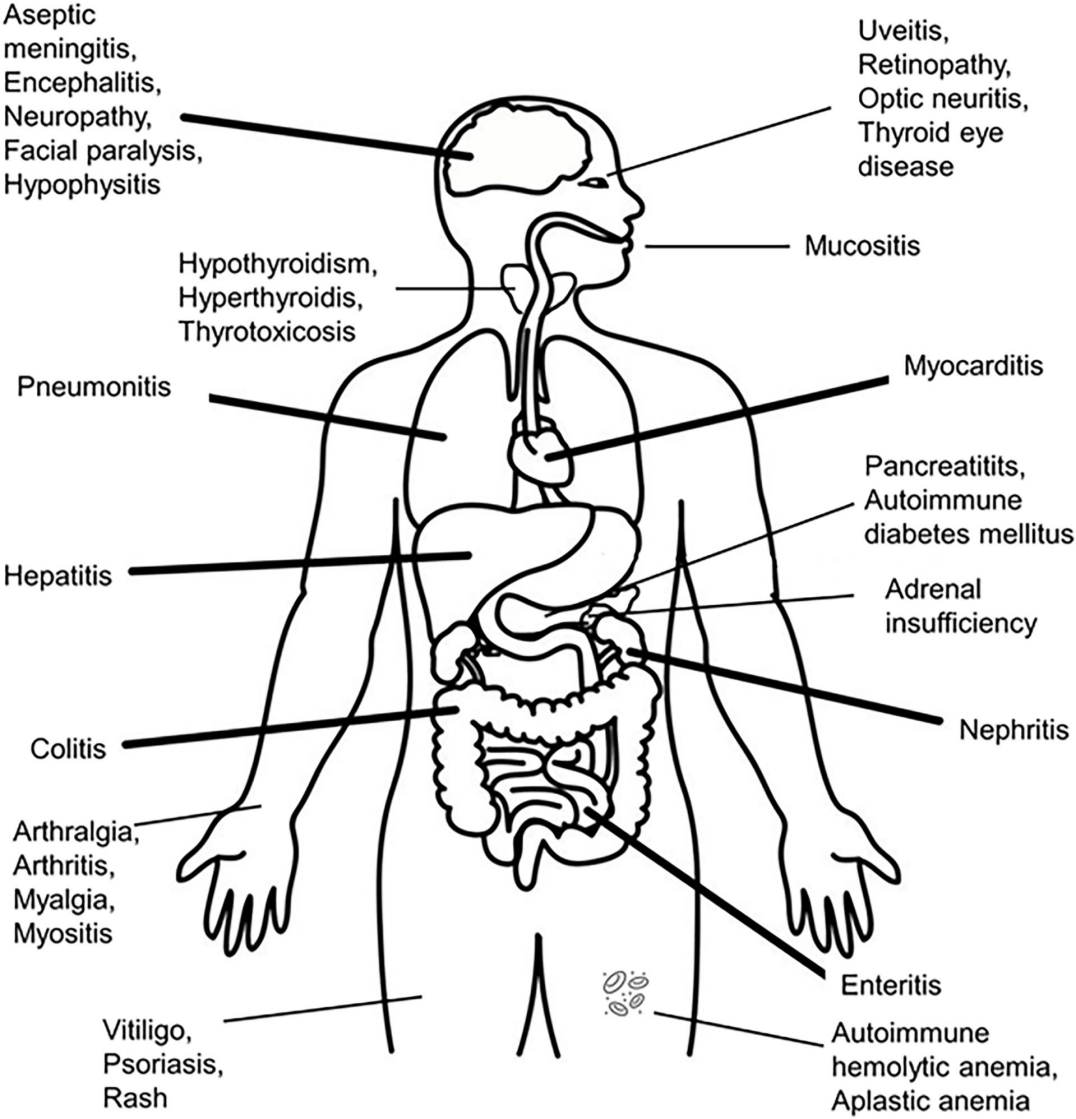
Organs suffering from immune-related adverse events after administration of immune checkpoint inhibitors.

IrAEs can involve the following sites: skin, gastrointestinal tract, liver, endocrine system, cardiovascular system, lungs, kidneys, pancreas, bone marrow, musculoskeletal system, nervous system, ocular system (see **Figure 2**). The endocrine related adverse events are the most frequent, as they are present in up to 40% of the patients treated with different ICIs – in descending order, the affected organs are: thyroid, hypophysis (both of these having a rich vascularization, ensuring contact with activated T-lymphocytes), adrenals, beta cells of the pancreatic islets (3, 68, 93–96) and parathyroid glands. Generalized lipodystrophy, autoimmune polyglandular syndrome (97, 98), hypercalcemia (99), low testosterone levels (in the absence of hypophysitis) and a single case of “suspicious” adrenocorticotropic hormone (ACTH)-independent cortisol secretion (in the absence of exogenous cortisol administration) after ICIs’ administration, have also been described (76, 100).

Although the pathogenesis is not clearly elucidated, there are many mechanisms that have been incriminated in irAEs development: genetic susceptibility which triggers autoimmunity, cross-reactivity of neoantigens released from tumoral and normal cells, or production of cytokines (101). In addition, different ICIs might involve different mechanisms and sites, seeing that the anti-CTLA-4 antibodies target the hypophysis more frequently, while the anti-PD-1 antibodies are usually linked to thyroid dysfunctions (9): this is also linked to the presence of CTLA-4 expressed in the pituitary gland [mostly on cells secreting prolactin or thyroid stimulating hormone (TSH)] and the expression of PD-1 and PD-L1 in the thyroid (68, 102).

Unfortunately, endocrinopathies induced by ICIs are usually irreversible (50% recovery of the pituitary-thyroid axis, 50-60% recovery of the pituitary-gonadal axis) (9, 62, 70, 103). They can occur at any time after the ICI administration. They usually tend to develop early after therapy initiation. However, seeing that the benefic, anti-cancer effects are still present after discontinuation of ICIs, there is also a risk of late development of adverse reactions (104, 105).

Risk factors for developing irAEs are the administration of high doses of ICI (only while using CTLA-4 inhibitors), preexisting autoimmune diseases (3, 106) and obesity (through the proinflammatory metabolic state) (107, 108). It is not known whether certain races may develop irAEs more frequently than others, which is why more studies need to be conducted on minorities and differentiated populations (101).

Clinicians should be vigilant in order to detect irAEs as early as possible. Before initiating treatment with ICIs, a few initial tests are recommended: thyroid function tests (TSH, free T4, free T3), hypophysis function tests (early morning cortisol levels), fasting glucose, adrenal function tests (ACTH), gonadal function tests (testosterone, luteinizing hormone – LH, and follicle-stimulating hormone – FSH), brain magnetic resonance imaging (MRI) (1, 93, 94). Patients should be monitored after each course during the first semester, every two courses over the next 6 months, and afterwards more rarely, as needed, considering the clinical suspicion (68, 109).

The management of the endocrine irAEs does not usually involve stopping the administration of ICIs (97, 103). Mild irAEs need low/moderate doses of steroids (0.5 mg/kg of prednisone or equivalent) and supportive therapy for 4-6 weeks, with the possibility of using other immunosuppressants the patient does not respond to steroids (3, 93).

Several research studies suggest that the occurrence of irAEs could predict a favorable response to treatment with ICIs, increased survival and progression-free survival, especially linked to PD-1 or PD-L1 inhibitors administration (5, 110).

**Hypophysitis** is the most frequent irAE found in patients treated with anti-CTLA-4 antibodies (9) (around 5% of the patients) (111), with higher incidence in patients treated with combination therapy involving ipilimumab and nivolumab, even though the mechanisms are not fully understood (96, 97). While hypophysitis generally affects women, ICI-related hypophysitis seems to be more frequently found in male patients. It tends to appear either within the first 2-3 months of therapy or even 19 months after (12, 112, 113). The supposedly direct proportional relationship between the doses of ipilimumab and the incidence of hypophysitis has been discussed in several studies, without a consensus being reached (64, 100, 114). Symptoms associated with hypophysitis are: fatigue, muscle weakness, headache, anorexia, nausea, weight loss, visual changes, intolerance of temperatures, arthralgias, mental status alteration. Hyponatremia, low ACTH or low TSH might be present (9, 100, 114). The suspicion of hypophysitis appears when two of the symptoms are identified, and the diagnosis is confirmed by hypophysis enlargement found on the brain MRI (which subsides after steroid treatment) (100, 111, 115). Given that the vast majority of the symptoms are unspecific, they can be overlooked, and the patient could develop life threatening complications. Usually, the hypopituitarism developed during hypophysitis is permanent and it does not respond to high doses of steroids (70, 97, 103), leading to infertility among other consequences (96, 100). Treatment with ICIs should not be discontinued, and the patient should receive replacing pituitary hormones (84).

**Immune checkpoint inhibitor-related diabetes mellitus** has been reported after treatment with PD-1/PD-L1 inhibitors (10, 12). Patients should be instructed regarding the associated symptoms of this irAE, in order to avoid life-threatening complications (93, 116). Diabetes mellitus can appear a few weeks after the ICI treatment initiation, or belatedly, after more than a year (97, 117), mostly in patients with no prior prediabetes, diabetes or other autoimmune diseases. However, islet autoantibodies have been identified in 50% of the patients that have been tested (10), and genetic susceptibility through HLA variants could be involved in diabetes development (fulminant type 1 diabetes mellitus was associated with HLA DRB_1*04:05-_ DQB_1*04:01_) (10, 117). The average blood glucose level at diagnosis is higher than 550 mg/dL (116, 118), the patients accusing polyuria, polydipsia, weight loss and sometimes nausea, vomiting, abdominal pain, lethargy or even coma if diabetic ketoacidosis occurs (119). The exocrine component of the pancreas could be affected, with increased lipase levels (120). Monitoring blood glucose is crucial for a prompt diagnosis. High-dose corticosteroids are not able to reverse the ICI-related diabetes mellitus (97, 121), therefore the treatment is centered on administering insulin analogs in order to maintain the glycated hemoglobin below 8% (68, 109), followed by long-term insulin therapy (10, 12, 81).

**Immune checkpoint inhibitor-related primary adrenal insufficiency** presents itself by elevated ACTH levels and low glucocorticoids and mineralocorticoids, usually installed 10 weeks after initiating the treatment with ICIs (97). It seems to be more frequent in men and after administration of ipilimumab, nivolumab and pembrolizumab. The patient can report fatigue, ortostatic hypertension and abdominal disconfort (12, 122). Tests should be conducted in order to differentiate primary adrenal insufficiency from secondary adrenal insufficiency (as the first requires both glucocorticoid and mineralocorticoid administration). In the case of adrenal crisis, the treatment should include high doses of glucocorticoids (100 mg hydrocortisone administered intravenously, followed by 50 mg hydrocortisone, every 6 hours) (10, 97).

**Immune checkpoint inhibitor-related primary hypoparathyroidism** involves acute hypocalcemia along with low levels of parathormone (68). In some cases, autoantibodies against the calcium-sensing receptor have been identified (123, 124). In most cases, the parathyroid dysfunction is irreversible, requiring continuous calcium and active vitamin D administration (97, 124).

**Acquired generalized lipodystrophy** represents the loss of subcutaneous fat alongside central obesity and insulin resistance after initiating treatment with ICIs; the level of leptin is low (97, 108).

**Autoimmune polyendocrine syndrome type II** has been stated as a rare endocrine irAE (125). It is defined by the presence of two out of the following three elements: autoimmune thyroid disease, type 1 diabetes mellitus and Addison’s disease (126). Mutations in CTLA-4 or HLA DR3-DQ2 and HLA DR4-DQ8 variants might increase the risk of developing this syndrome after the administration of ICIs (125, 127).

## Thyroid-related adverse events linked to immune checkpoint inhibitors

Thyroid dysfunctions, such as hypothyroidism, thyrotoxicosis, painless thyroiditis or the “thyroid storm” are present in 1-6% of the patients treated with ICIs (9, 114, 128) (other authors suggest the percentage amounts to over 10%) (86). Painless thyroiditis is probably under-reported, since it is not clinically suggestive, while hypothyroidism ranges between 1-15% and hyperthyroidism between 1-10%, in ICI-treated patients (pembrolizumab alone, nivolumab alone or combinations) (6, 53, 70, 92, 129).

There are certain risk factors linked to the development of thyroid-relates irAEs after ICI administration: gender (females seem to have a higher risk), age (younger patients are at risk) (97, 130), race (Caucasian and Hispanic races are associated with hypothyroidism, while African Americans are linked to thyrotoxicosis) (101), elevated anti-thyroid peroxidase antibodies (TPOAb) or anti-thyroglobulin-antibodies (TgAb) (27, 131), higher baseline TSH, a higher number of treatment cycles, usage of PD-1 and PD-L1 inhibitors rather than CTLA-4 inhibitors, obesity (linked to earlier occurrence of thyroid irAEs – every increase of 1 kg/m^2^ of the body mass index rises the risk of symptomatic thyrotoxicosis to 10%) (107, 132), increased fluorodeoxyglucose (^18^FDG) uptake in the thyroid before initiating treatment (65, 97), and renal cell carcinoma (probably due to the fact that before treatment with ICI, sunitinib is usually used for treating these patients and it can affect thyroid function as well) (133).

The thyroid irAEs usually develop 6 weeks after starting the treatment (3, 134), but they can also appear years after treatment initiation (19, 135, 136); therefore, frequent monitoring of patients treated with ICIs is important even after therapy completion (137). ICI combinations are linked to earlier development of thyroid dysfunctions (135, 136).

Many theories have emerged regarding the pathogenesis of thyroid-related irAEs. However, the most accepted current theory involves an interplay between genetic factors, cellular autoimmunity and humoral immunity, supported by T-cells cross-reactivity, increased levels of interferon gamma-inducible chemokines (which attract T-cells), the contribution of ADCC and the HLA-DR allele which is involved in autoimmunity (5, 138). Anti-PD-1 antibody-induced thyroiditis seems to be primarily a T-cell mediated process, supported by the presence of CD8^+^ T-cells in the thyroid, and CD4^-^CD8^-^ T-cells in the thyroid and blood of the patients (139, 140). However, T-cell dependent activation of B-cells has been reported, resulting in production of autoantibodies which could serve as biomarkers for ICI-induced immunogenicity and therapeutic response (141). A modified Th1/Th2 balance has been reported in favor of Th1, with increased levels of IL-2 (which might stimulate autoreactive lymphocytes), IL-1β, GM-CSF and decrease of Il-8, G-CSF and MCP-1 (111, 142).

Two clinical patterns of thyroid dysfunction related to the administration of ICIs have been described: 1. hyperthyroidism, followed by hypothyroidism; 2. *de novo* hypothyroidism. There is also the triphasic pattern of presentation in some of the patients (hyperthyroidism, followed by hypothyroidism and euthyroidism), while the remaining patients experience isolated hypothyroidism or hyperthyroidism, followed by euthyroidism (88, 143). However, the distinction between hypo-, hyper- and euthyroidism should not be radical, seeing that they are part of the same process (129). Another classification of thyroid irAEs has been made regarding the duration of therapy needed for managing the thyroid dysfunction – there are thyroid irAEs which need continuous therapy for thyroid dysfunction (linked to maximum levels of thyroglobulin and thyroglobulin antibodies while administering ICIs) and those in need of temporal treatment only (144).

The patients with thyroid irAEs developed after treatment with ICIs can have three different clinical presentations: 1. newly onset hypothyroidism (TSH ≥ 4.3 mIU/L, free T4 ≤ 0.8 ng/dL) or subclinical hypothyroidism (TSH ≥ 4.3 mIU/L, free T4: 0.9-1.7 ng/dL); 2. symptomatic (overt) thyrotoxicosis (TSH ≤ 0.2 mIU/L, free T4 ≥ 1.8 ng/dL) or subclinical hyperthyroidism (TSH ≤ 0.2 mIU/L and free T4: 0.9-1.7 ng/dL); 3. acute elevation of TSH in patients previously diagnosed with hypothyroidism, requiring a 50% increase of the levothyroxine dose (145). Low levels of TSH accompanied by low or normal levels of free T4 and free T3 appear in the context of euthyroid sick syndrome, which is a differential diagnosis for thyroid irAEs (146).

Patients can be asymptomatic, or they might show nonspecific symptoms, such as tachycardia or palpitations (11, 103). However, there are 5 grades of severity for thyroid irAEs according to CTCAE, similar to all the other endocrine adverse events related to ICI administration: grade 1 – mild, grade 2 – moderate, grade 3 – severe, but not immediately life-threatening, grade 4 – life-threatening, grade 5 – death (see **Table 2** for the general criteria) (87).

The thyroid workup involves measuring TSH, free T4 (which seems to successfully predict recovery, because it is less sensitive than TSH to variations of the thyroid hormone feedback), and also free T3 can be measured in cases of thyrotoxicosis (93, 94, 147).

Studies have linked irAEs to improved survival in cancer patients treated with ICIs (148, 149). Therefore, it was hypothesized that thyroid dysfunction could be seen as a predictive biomarker of treatment response (139) (patients with acute symptomatic thyrotoxicosis seem to have a better prognosis than patients without thyroid irAEs) (139, 145, 150). The presence of thyroglobulin antibodies has also been correlated with increased survival (as they are markers of a potent activation of the immune system) (151). Variations of thyroid volume might serve as imagistic markers of therapeutic response as well (due to the increase in volume after administration of a PD-1 inhibitor) (152).

### Thyrotoxicosis and hyperthyroidism

“Thyrotoxicosis” refers to the excess of thyroid hormones of any cause, while “hyperthyroidism” is a more specific term, referring to an excess of thyroid hormones caused only by overactivity of the thyroid. Despite the terminology, many studies use both words as equivalents, leading to an underestimation of ICI-triggered Graves’ disease (which results in hyperthyroidism) (5). Hyperthyroidism is less common than hypothyroidism among patients under ICI treatment, with an incidence of 1-7% (6, 53, 70, 92), which is probably underestimated. CTLA-4 inhibitors cause hyperthyroidism in 0.2-1.7% of the patients, while PD-1/PD-L1 inhibitors affect 0.6-3.7% and combinations of ICIs are responsible for 8-11% of the cases (96, 97, 153).

Hyperthyroidism usually appears after 21 days of combination therapy, or after 47 days when a PD-1 inhibitor is administered alone (8, 129, 153). Graves’ disease induced by ICI appears mostly at the beginning of the treatment. However, in some cases it can occur many weeks later (90). After 3-6 weeks of the initial phase of thyrotoxicosis, hypothyroidism is frequently developed (96, 136).

The pathogenesis is variable, hyperthyroidism/thyrotoxicosis may occur either in the context of thyroiditis (when thyroid follicles are destroyed and the hormones are spilled into the bloodstream), or because of autoantibody development (e.g. Graves’ disease) (3, 6). ICI-induced thyroiditis is considered a T-cell mediated process, with CD8^+^ and CD4^-^CD8^-^ T-cells found in the thyroid (140), as well as clusters of necrotic cells and CD163^+^ histiocytes (154).

The patients usually present with palpitations, heat intolerance, tremor, anxiety, emotional lability, weight loss in the presence of increased appetite, atrial fibrillation, hyperdefecation, oligo/amenorrhea in women and erectile dysfunction in men (5, 155). A phenomenon called ‘thyroid storm’ can sometimes occur, which involves fever, tachycardia or atrial fibrillation, signs of congestive heart failure and diarrhea, which poses a great threat (97, 156, 157).

When the clinician observes these symptoms, thyroid function tests should be recommended: the patient suffering from hyperthyroidism will have low levels of TSH and a high free T4 (96). Afterwards, thyroid autoantibodies should be measured (97) (pembrolizumab increases anti-thyroid peroxidase antibodies and anti-thyroglobulin antibodies in 80% of the patients with ICI-induced thyroid dysfunction) (158). Thyroid ultrasound (which can distinguish Graves’ disease from thyroiditis by vascularity), scintigraphy, technetium or iodine scanning, positron emission tomography scan (PET) might also be used for differential diagnosis. Increased uptake of ^18^FDG on PET scan is suggestive for destructive thyroiditis (65, 96, 97).

Graves’ disease which developed after treatment with ICIs, consists of hyperthyroidism, diffuse goiter and sometimes ophthalmopathy (159, 160). However, there are also cases of ophthalmopathy in the absence of elevated thyroid hormones (161–163). This clinical entity has been named thyroid eye disease (TED)-like orbital inflammatory syndrome, and it involves eye pain, conjunctival redness, periorbital edema, proptosis and ophthalmoplegia (5, 160). Considering that allelic variants of CTLA-4 are involved in Graves’ disease development, CTLA-4 and PD-1 gene polymorphisms might be involved in the occurrence of TED (21, 164–166). 60-70% of the Graves’ disease cases evolve over a long period of time, with numerous relapses and remissions, while 30-40% of the patients have only one episode of hyperthyroidism. It is possible that patients experience spontaneous remission (5, 167, 168).

### Hypothyroidism

Hypothyroidism is the most frequent manifestation of thyroid irAEs, and it usually occurs 6 weeks after subclinical hyperthyroidism, in the context of destructive thyroiditis (in 30-40% of the patients treated with anti-PD-1/PD-L1 antibodies). Therefore, patients diagnosed with ICI-related thyroiditis should be monitored for the development of hypothyroidism (3, 6, 134, 145). CTLA-4 inhibitors cause hypothyroidism in 2.5-5.2% of the patients, while PD-1/PD-L1 inhibitors affect 3.9-8.5% and combinations of ICIs are responsible for 10.2-16.4% of the cases (96, 97, 153). Hypothyroidism usually occurs ~63 days after initiation of ICI-combination treatment, and ~70 days after intake of PD-1 inhibitors alone. Risk factors do not include age or gender (8, 155). However. they comprise preexistent autoimmune diseases (e.g. type 1 diabetes mellitus, gastric atrophy) and certain HLA-DR alleles (1, 65, 169).

The debut is often nonspecific, especially when hypothyroidism is classified as a grade 1 or 2 (97), the patients presenting weight gain, fatigue, cold intolerance, constipation, dryness of the skin, bradycardia, periorbital edema and tongue swelling (10, 100, 153, 155). The TSH levels are high, while free T4 is decreased (97). Rarely, hypothyroidism-associated myositis might occur (described in a patient after administration of nivolumab), which involves severe myalgia, arthralgia and high levels of creatine kinase (170, 171). Fortunately, this condition is reversible after levothyroxine replacement therapy. Myxedema crisis was also reported in a few cases (11, 97, 172).

It is important that patients who suffer from preexistent hypothyroidism are closely monitored with thyroid function tests at all times after initiation of ICis in order to adjust the levothyroxine doses (10, 65). Hypothyroidism developed as irAE is oftentimes permanent and it requires levothyroxine replacement therapy (10, 97).

## Management of thyroid adverse events linked to immune checkpoint inhibitors

The American Society of Clinical Oncology (ASCO) has established a set of guidelines for performing the thyroid function tests: TSH and free T4 should be monitored every 4-6 weeks from starting the ICIs. TSH is considered to be the optimal screening test, accompanied by free T4 when its values are modified (93).

The treatment with ICIs should not be interrupted if thyroid irAEs occur, except when severe symptoms occur and in cases of TED, when ICIs are stopped until the symptoms disappear (8, 93, 97). Severe irAEs also require administration of steroids in order to limit the inflammation, or infliximab (anti-TNFα antibody), mycofenolate mofetil, tacrolimus, cyclosporine in cases of lack of response to steroids (6, 173). The endocrinopathies developed after ICI therapy are generally managed by the treating oncologists, but collaborations with endocrinologists should be considered in complicated or more severe cases (9, 103).

### Management of thyrotoxicosis and hyperthyroidism

Prevention is key for avoiding severe irAEs. Therefore, monitoring the patient through thyroid function tests is very important throughout the treatment with ICIs (174): testing should be performed before therapy initiation, and after every 2 months (9, 93). Ultrasonographic monitoring could be useful in decision-making, considering that variations of thyroid volume related to the thyroid dysfunction have been discovered (first, the thyroid grows in volume after initiation of ICI treatment, and afterwards it starts shrinking, indicating the need of continuous thyroid hormone replacement after stopping the ICIs) (175).

Treatment options are chosen considering the CTCAE grading. Because hyperthyroidism is most frequently of grade 1 or 2 (mild or moderate), ICI therapy does not usually need to be interrupted and patients should only receive symptomatic treatment with beta blockers (8, 9, 93). If the patient is asymptomatic, she/he should only be observed and monitored thoroughly until symptoms develop, TSH decreases (5-10 mIU/L or lower) or TPO antibodies increase (93, 97). ICIs should be interrupted if the patient suffers from a grade 3 thyroid irAE and, in these cases, in general, corticotherapy should be initiated: 1-2 mg/kg/day of oral prednisolone. In grade 4 hyperthyroidism, corticotherapy is needed and it consists of 1-2 mg/kg/day intravenous methylprednisolone first, followed by oral prednisolone (1-2 mg/kg/day), decreasing the dose after one month (9, 93). However, some studies have indicated that doses of prednisone (or equivalent) higher than 7.5 mg/day have not shown benefits compared to no administration of glucocorticoids whatsoever, in terms of the duration of thyrotoxicosis, time until onset of hypothyroidism and dose of levothyroxine needed for hormonal replacement (5, 176). Depending on whether the patient suffers from thyrotoxicosis in the context of destructive thyroiditis or hyperthyroidism in the context of Graves’ disease, anti-thyroid therapy with methimazole or propylthiouracil might be considered (it is only effective for hyperthyroidism, and not for the thyrotoxicosis caused by thyroiditis) (93, 97). Therefore, patients developing Graves’ disease after the administration of ICIs should receive anti-thyroid drugs (which are usually able to maintain the euthyroidism), and only if the hyperthyroidism is not manageable through drug administration, radioiodine or thyroidectomy should be considered (158, 177). There is also a chance of spontaneous remission of hyperthyroidism caused by Graves’ disease, even if no treatment is administered (5, 168). While speaking about TED developed in the context of ICI treatment, there might be mild cases which are usually treated topically (with artificial tears, for instance), but also dramatic cases which require high doses of steroids that are usually able to stop the inflammation and lead to symptoms resolution (84, 160).

### Management of hypothyroidism

For early detection of hypothyroidism as an irAE developed after ICI treatment, ASCO recommends measuring TSH and free T4 every 4–6 weeks after initiation of treatment, while also monitoring the patient after stopping the ICIs (93), while the European Society for Medical Oncology (ESMO) additionally proposes screening before starting therapy (measuring TSH, free T4 and free T3) (94).

Serum TSH levels determine what doses of levothyroxine are needed for thyroid hormonal replacement therapy. When TSH is mildly elevated (5-10 mIU/L), hormonal replacement therapy can be initiated, with low doses of levothyroxine (25-50 µg/day). If the patient is an elder with cardiovascular comorbidities, lower doses should be considered (12.5-25 µg/day), while a young, healthy patient could receive doses of 1.6 µg/kg (full estimated replacement dose) from the start (84, 97, 178). However, a recent research study has shown that in a murine model of lung carcinoma, levothyroxine increased tumor growth, while the administration of liothyronine, which is a form of T3, has prolonged survival by suppressing tumor growth. Therefore, more studies regarding the use of liothyronine instead of levothyroxine for hormonal replacement in the context of neoplasia should be conducted (179).

## Discussion and conclusion

While cancer is on the rise, novel treatments are required for a better management of the disease. ICIs are a new class of drugs, emerged in the last decade, which show promising results and increased survival in cancer patients. Since ICIs are classified as a subtype of immunotherapy, their main mechanism is removing the brakes on the immune system, thus facilitating the destruction of the malignant cells. This comes bundled with a new category of adverse reactions, known as irAEs, which can affect the whole body through autoimmunity. Endocrine irAEs are particularly found in patients treated with ICIs, and among them the thyroid irAEs (including hyperthyroidism, thyrotoxicosis, hypothyroidism, ‘thyroid storm’) are the most frequent.

At this time, the exact mechanisms of thyroid irAEs induction by each of the ICIs is not fully understood. Many theories have emerged, and the most accepted current theory involves an interplay between genetic factors, cellular autoimmunity and humoral immunity, supported by T-cells cross-reactivity, increased levels of interferon gamma-inducible chemokines (which attract T-cells), the contribution of ADCC and the HLA-DR allele which is involved in autoimmunity (5, 138). The PD-1 inhibitors such as pembrolizumab, nivolumab and cemiplimab, are IgG4 antibodies and do not activate classical complement pathway, thus having less potency to initiate ADCC. Anti-PD-1 antibody-induced thyroiditis seems to be primarily a T-cell mediated process, supported by the presence of CD8^+^ T-cells in the thyroid, and CD4^-^CD8^-^ T-cells in the thyroid and blood of the patients (139, 140). However, T-cell dependent activation of B-cells has been reported, resulting in production of autoantibodies which could serve as biomarkers for ICI-induced immunogenicity and therapeutic response (141). A modified Th1/Th2 balance has been reported in favor of Th1, with increased levels of IL-2 (which might stimulate autoreactive lymphocytes), IL-1β, GM-CSF and decrease of Il-8, G-CSF and MCP-1 (111, 142). In contrast, PD-L1, such as atezolizumab, durvalumab and avelumab and anti-CTLA-4 inhibitors, such as ipilimumab, activate the classical complement pathway and initiate the ADCC.

While all of the ICIs have somewhat overlapping thyroid irAEs, PD-L1 inhibitors, such as atezolizumab and avelumab, have a predilection in inducing hypothyroidism, while the PD-1 and CTLA-4 inhibitors can cause both hypothyroidism and hyperthyroidism (**Table 1**). CTLA-4 inhibitors cause hyperthyroidism in 0.2-1.7% of the patients, while PD-1/PD-L1 inhibitors affect 0.6-3.7% and combinations of ICIs are responsible for 8-11% of the cases (96, 97, 153). Anti-CTLA-4 antibodies are known to have hypophysitis as the most frequent irAE (9) (around 5% of the patients) (111). Immune checkpoint inhibitor-related diabetes mellitus has been reported after treatment with PD-1/PD-L1 inhibitors (10,12, **Table 1**).

A major inconvenience is that many thyroid irAEs developed in the context of ICI therapy are irreversible and in need of long-term hormonal replacement. However, there is a positive side: thyroid irAEs have been associated with better cancer outcomes, with increased survival and better prognosis for the patient developing these adverse reactions. This is probably due to efficient activation of the immune system. A better understanding of the irAEs pathogenesis through further studies is required.

Monitoring the thyroid irAEs is firstly important for preventing the life-threatening situations that might occur, such as thyroid storm, and secondly as a means of overseeing treatment response and estimating the prognosis of the patient. A close collaboration between the treating oncologist and an endocrinologist is crucial, and also raising awareness of the existence of these adverse reactions should be a priority among both clinicians and patients, as the former could recognize early symptoms of hyper- or hypothyroidism and might seek help from the medical community sooner, receiving an optimal treatment and increasing their quality of life.

## Author contributions

AC and OB designed and organized the review. AC analyzed, summarized the information, and wrote the manuscript. AS contributed to the writing and improvement of the review. OB supervised the work and contributed to the writing and improvement of the manuscript. All authors contributed to the article and approved the submitted version.

## Funding

OB is funded by a grant of the Romanian Ministry of Education and Research, CNCS - UEFISCDI, project number PN-III-P4-ID-PCE-2020-2027, within PNCDI III. Authors would also like to acknowledge the funding from Ministry of Research, Innovation and Digitization in Romania, under Program 1 – The Improvement of the National System of Research and Development, Subprogram 1.2 – Institutional Excellence – Projects of Excellence Funding in RDI, Contract No. 31PFE/30.12.2021.

## Acknowledgments

Authors would like to acknowledge the extraordinary environment and support from our host institutions.

## Conflict of interest

The authors declare that the research was conducted in the absence of any commercial or financial relationships that could be construed as a potential conflict of interest.

## Publisher’s note

All claims expressed in this article are solely those of the authors and do not necessarily represent those of their affiliated organizations, or those of the publisher, the editors and the reviewers. Any product that may be evaluated in this article, or claim that may be made by its manufacturer, is not guaranteed or endorsed by the publisher.
